# Methionine Protects Mammary Cells against Oxidative Stress through Producing S-Adenosylmethionine to Maintain mTORC1 Signaling Activity

**DOI:** 10.1155/2021/5550196

**Published:** 2021-07-19

**Authors:** Heju Zhong, Peiqiang Yuan, Yunxia Li, Dolores Batonon-Alavo, Caroline Deschamps, Bin Feng, Xiaoling Zhang, Lianqiang Che, Yan Lin, Shengyu Xu, Jian Li, Yong Zhuo, Gang Tian, Jiayong Tang, Xuemei Jiang, Lingjie Huang, Caimei Wu, De Wu, Zhengfeng Fang

**Affiliations:** ^1^Key Laboratory for Animal Disease Resistance Nutrition of the Ministry of Education, Animal Nutrition Institute, Sichuan Agricultural University, Chengdu 611130, China; ^2^Adisseo France SAS, F-03600 Commentry, France

## Abstract

The mechanistic target of rapamycin complex 1 (mTORC1) signaling plays pivotal roles in cell growth and diseases. However, it remains mechanistically unclear about how to maintain mTORC1 activity during mammary glands development. Here we showed that mammary glands suffered from aggravated oxidative stress as pregnancy advanced and was accompanied by an increase in H_2_O_2_ levels, while the consumption for methionine and *S*-adenosylmethionine (SAM) rather than *S*-adenosylhomocysteine (SAH) were promoted *in vivo*. Likewise, H_2_O_2_ promoted SAM synthesis and reduced SAM utilization for methylation depending on H_2_O_2_ levels and treatment time *in vitro*. H_2_O_2_ inhibited phosphorylation of S6 kinase Thr 389 (p-S6K1 (T389)), 4E-BP1 Thr 37/46 and ULK1 Ser 757, the downstream of mTORC1, in mammary epithelial cells. However, methionine and SAM were shown to activate mTORC1 under H_2_O_2_-exposed condition. Moreover, this effect was not disabled by SGI-1027 which inhibits SAM transmethylation. In conclusion, methionine appeared to protect mammary cells against oxidative stress through producing SAM to maintain mTORC1 signaling activity.

## 1. Introduction

While the physiological level of reactive oxygen species (ROS) like hydrogen peroxide (H_2_O_2_) is essential to regulate many normal cellular processes [1], excess ROS production has been linked to over 150 diseases including tumorigenesis, diabetes mellitus, atherosclerosis, and neurodegenerative diseases [2]. Notably, oxidative stress not only causes oxidative damage, but also induces autophagic cell death [3, 4], thus not conducive to normal organ development.

Mammary glands, as a unique model to study organ specificity and development [5], have triggered many studies demonstrating that ROS can lead to mammary dysplasia or even make mammary dysplasia progress to breast cancers [6]. However, it is far from being completely understood why mammary glands usually achieve rapid development despite the increased oxidative stress as manifested by enhanced DNA damage while markedly decreased vitamin A and E levels, with the advance of pregnancy in both women [7] and sows [8]. Therefore, to uncover the metabolic and molecular mechanism for developing mammary glands to withstand oxidative stress might provide a new theory basis for developing the interventions and preventive strategies for oxidative stress in animal and human disease.

Mammary gland development refers to a lot of anabolic biological processes, such as DNA and protein synthesis, accompanied by proliferation of mammary epithelial cells. The mechanistic target of rapamycin complex 1 (mTORC1) signaling pathway has emerged as a key pathway to trigger cell proliferation and growth through phosphorylating 4E-BP1 and S6 kinase 1 (S6K1) [9]. Oxidative stress has been shown to inhibit the mTORC1 signaling pathway [4]. A recent study indicates that SAM can dissociate SAMTOR-GATOR1 complex by binding directly to SAMTOR to activate mTORC1 [10]. This leads us to hypothesize that methionine might reduce oxidative stress through SAM-mediated mTORC1 activation, independently to the transmethylation required by methionine metabolism to synthesize antioxidants such as glutathione and taurine. To test our hypothesis, we first used the mammary glands before and after rapid cell proliferation as the model to explore the dynamic change of tissue-specific levels of methionine, SAM and *S*-adenosylhomocysteine (SAH), the oxidative status as indicated by extracellular H_2_O_2_ levels, the expression of proteins to reflect mTORC1 signaling activation, and the change of biological processes through the metabolomics relative-quantitative analysis. Then, we used primary mammary epithelial cells to further determine whether methionine and its non-transmethylation product SAM can activate mTORC1 signaling under oxidative stress exposed to the inhibitor that prevents methionine and SAM transmethylation metabolism.

## 2. Materials and Methods

All experiment procedures were approved by Animal Care and Use committee of Animal Nutrition Institute, Sichuan Agricultural University.

### 2.1. Animal Studies

Given that late gestation is a critical period for sow mammary gland development, 12 primiparous sows (Landrace × Yorkshire) were used for experiment from pregnancy 60 to 90 days. The pregnant diet was formulated according to National Swine Nutrition Guide (2010), and the daily intake of nutrients, including 6.99 g/d methionine and 15.82 g/d sulfur amino acids, was the same for each sow during experiment. About 93% and 7% of methionine was from dietary basal ingredients and crystalline DL-methionine, respectively. Six primiparous sows on 60 and 90 days of pregnancy, respectively, were selected for sample collection. Mammary extracellular fluid in the left third mammary gland was collected using microdialysis method under fasting conditions on 60 and 90 days of pregnancy, respectively, and the specific operation referred to the methods we previously described in detail [11]. Mammary parenchymal tissues were collected immediately after animals were sacrificed. The cross-sectional area of right individual mammary parenchymal tissue was measured using graph paper, and their arithmetic mean was used as representative data for each animal.

### 2.2. Cell Culture

The cell culture method referred to previous reference [12]. Primary explant culture method was used to obtain cells, and the 0.25% trypsin-EDTA (Gibco) was used to remove fibroblasts and purify mammary epithelium cells. The purity of mammary epithelium cells was identified using flow cytometer (BD Biosciences) with keratin 18 antibody (Bioss), and mammary epithelial cells accounted for more than 94% of the total (Supplementary Figure 1E). Mammary epithelium cells were maintained in DMEM/F12 medium supplemented with 10% fetal bovine serum (Gibco), 5 *μ*g/mL insulin (Sigma-Aldrich), 1 *μ*g/mL hydrocortisone (Sigma-Aldrich), 5 ng/mL recombinant swine epidermal growth factor (Kingfisher Biotech) and Antibiotic-Antimycotic (Gibco) at 37°C with 5% CO_2_. There were at least 3 replicates in each experiment.

By determining the sensitivity of mTORC1 activation to methionine within mammary epithelium cells, we found that methionine starvation hardly affected mTORC1 activity less than 2 h (Supplementary Figure 2A), and low concentrations (10-20 *μ*M) of methionine could satisfy mTORC1 activation within 4 h (Supplementary Figure 2B). And p-S6K1 (T389) was more sensitive to mTORC1 activation (Supplementary Figure 2A) [13]. Therefore, methionine-free medium was used for less than 2 h in the experiment, and the optimal methionine concentration, 67 *μ*M, under physiological conditions (26-68 *μ*M in mammary vein), was selected in the experiment according to regression analysis (Supplementary Figure 2B). H_2_O_2_ concentrations in mammary tissues determined at pregnancy day 60 and 90 were among 0.1-1.8 mM. The decreased cell viability was observed at 0.4 mM H_2_O_2_, thus 0.4 mM H_2_O_2_ was used in cell viability experiments. The inactivated mTORC1 signaling was observed at 0.8 mM H_2_O_2_, therefore 0.8 mM H_2_O_2_ was used in cell signaling experiments. In experiments regarding redox homeostasis, metabolism, time- and dose-dependent effects of H_2_O_2_ on mTORC1 signaling pathway, mammary epithelial cells were incubated in the DMEM/F12 basic medium 2 h before H_2_O_2_ treatment, and then were incubated in the DMEM/F12 basic medium during treatment. For short-term (<24 h) trails involving the addition of methionine or HMTBA, mammary epithelial cells were incubated in the DMEM/F12 basic medium 2 h before methionine or HMTBA treatment, and then were incubated in the methionine-free DMEM/F12 basic medium during treatment. The SGI-1027 (Selleck) is a methyltransferase inhibitor [14]. For SGI-1027 experiment, according to the pre-experiment, a short time of inhibitor treatment did not change the expression of p-s6k1 (T389), thus, methyltransferase-off mammary epithelial cells were treated with SGI-1027 for 48 h. Later, mammary epithelial cells were incubated in the DMEM/F12 basic medium 2 h before methionine or HMTBA treatment, and then were incubated in the DMEM/F12 basic medium during treatment. Ademetionine 1,4-butanedisulfonate (Yuanye Bio-Technology) (SAMe) was used as a source of *S*-adenosylmethionine (SAM) in our experiment.

### 2.3. Hydrogen Peroxide Levels Analyses

Hydrogen peroxide concentrations in mammary dialysis fluid and tissues were determined using a commercial kit (Solarbio).

### 2.4. Histological Procedure

The left fourth mammary parenchymal tissues were determined using H&E stain method as previously described [15].

### 2.5. Mammary Chemical Analyses

The right side mammary parenchymal tissues were ground after weighing, and chemical composition was determined as previously described [15].

### 2.6. SAM and S-Adenosylhomocysteine (SAH) Analyses

The left fourth mammary parenchymal tissues were homogenized in ultrapure water, then they were centrifuged at 14000 r/min for 20 min at 4°C. Four hundred microliter supernatant and 40 *μ*L trichloroacetic acid solution (400 g/L) were mixed and centrifuged at 14000 g for 20 min at 4°C, and supernatant was collected. As for cell samples, cells were washed with cold PBS and lysed using cold lysis buffer (Beyotime), and supernatant was collected after centrifuging (12000 g for 30 min at 4°C). The protein concentration of supernatant was determined using BCA kit (Thermo Fisher Scientific). Supernatant was mixed with 10% (w/v) sulfosalicylic acid and centrifuged at 20000 g for 30 min at 4°C, and then supernatant was collected. The SAM and SAH levels in tissue and cell samples were determined using ultra performance liquid chromatography (UPLC, Waters) containing Waters ACQUITY UPLC BEHC18 column (150∗2.1 mm, 1.7 *μ*m) [16]. The SAM and SAH standards were purchased from Cayman Chemical (#13956) and Sigma-Aldrich (#A9384), respectively. The mobile phase with pH 3.1 contained 40 mM NaH_2_PO_4_, 8 mM sodium heptane sulphonate and 18% (v/v) methanol.

### 2.7. Methionine Determination

Sample preparation referred to SAM and SAH analyses. Methionine levels in samples were determined using High-Speed Amino Acid Analyzer LA8080 (Hitachi).

### 2.8. Untargeted Metabolomics Relative-Quantitative Analysis

The left fourth mammary parenchymal tissues (80 mg) were homogenized in ultrapure water (200 *μ*L). The 800 *μ*L methanol/acetonitrile (1 : 1, v/v) was added into homogenate, then they were sonicated at low temperature after mixing. Samples were incubated at -20°C for 1 h, then centrifuged for 20 min (14000 g, 4°C). The supernatant was dried in a vacuum centrifuge. For LC-MS/MS analysis, the samples were re-dissolved in 100 *μ*L acetonitrile/water (1 : 1, v/v) solvent. Analyses were performed using an UHPLC (1290 Infinity LC, Agilent Technologies, USA) coupled to a quadrupole time-of-flight (AB Sciex TripleTOF 6600) in Shanghai Applied Protein Technology Co., Ltd. The raw data were converted to MzXML files using ProteoWizard, before analyzing by XCMS software. In the extracted ion features, only the variables having more than 50% of the nonzero measurement values in at least one group were kept. Compound identification of metabolites by MS/MS spectra with an in-house database established with available authentic standards. After normalized to total peak intensity, the processed data were uploaded into before importing into SIMCA-P (version 14.1), where it was subjected to orthogonal partial least-squares discriminant analysis (OPLS-DA). The variable importance in the projection (VIP) value of each variable in the OPLS-DA model was calculated to indicate its contribution to the classification. Metabolites, the VIP value >1, were further applied to *t* test, and the *P* <0.05 were considered as statistically significant. Clustering and correlation of statistically significant metabolites were conducted in Metaboanalyst 4.0 software (http://www.metaboanalyst.ca). Distance measure using Euclidean, and clustering algorithm using Ward.D. Person's correlation was concerned in calculation. Statistically significant metabolites were blasted against the online Kyoto Encyclopedia of Genes and Genomes (KEGG) database (http://geneontology.org/) to retrieve their COs and were subsequently mapped to pathways in KEGG11. Then, the corresponding KEGG pathways were extracted.

### 2.9. Western Blot

The western blot analysis was conducted as previously described [17]. The left fourth mammary parenchymal tissues and mammary epithelial cells were lysed with cold lysis buffer (Beyotime), centrifuged at 12000 g for 30 min at 4°C, and the supernatant were mixed with loading buffer (Bio-Rad) and mercaptoethanol and boiled for 5 min. Samples were separated by SDS-PAGE with 10% or 15% acrylamide gel and transferred to PVDF membranes. Primary antibodies were Phospho-p70 S6 Kinase (Thr389) (#9205, 70 kDa, 1 : 1000, Cell Signaling Technology), p70 S6 Kinase (#9202, 70 kDa, 1 : 1000, Cell Signaling Technology), Phospho-4E-BP1 (Thr37/46) (#9459, 15-20 kDa, 1 : 1000, Cell Signaling Technology), 4E-BP1 (#9452, 15-20 kDa, 1 : 1000, Cell Signaling Technology), Phospho-ULK1 (Ser757) (#14202, 140-150 kDa, 1 : 1000, Cell Signaling Technology), ULK1 (#8054, 150 kDa, 1 : 1000, Cell Signaling Technology), Beclin-1 (#3495, 60 kDa, 1 : 1000, Cell Signaling Technology), LC3A/B (#12741, LC3A/B-I: 16 kDa, LC3A/B-II: 14 kDa, 1 : 1000, Cell Signaling Technology), PCNA (#2586, 36 kDa, 1 : 2000, Cell Signaling Technology) and *β*-actin (#4967, 45 kDa, 1 : 1000, Cell Signaling Technology). The secondary antibodies were purchased from Beyotime. The protein bands were quantified by Image Lab (Bio-Rad).

### 2.10. Malonaldehyde (MDA), Reduced Glutathione (GSH) and Glutathione Disulfide (GSSG) Assays

Cells were lysed using cold lysis buffer (Beyotime), and the concentrations of MDA, GSH and GSSG were measured using a commercially available kit (Beyotime).

### 2.11. Cell Viability Assays

Cells were seeded at a concentration of 2000 cells per well for 24 h in 96 well plates in 100 *μ*L complete medium. Then complete medium was removed from cell plates following compound treatment for 24 h in serum free medium. The cell counting kit-8 (Dojindo) was used to determine cell viability.

### 2.12. Statistical Analyses

Except for metabolomics data, others were analyzed using SAS 9.4 (SAS Institute). The TTEST procedure was used to analyze pregnant data if data were normally distributed. If the data was not normally distributed, the GLIMMIX procedure was used to analyze data, and the gamma distribution was chosen. The MIXED procedure was used to analyze data in multiple groups, the LSD method was used to conduct multiple comparisons, and orthogonal polynomial contrast was carried out in methionine requirement of mTORC1 signaling activation experiment. If the residuals were not normally distributed and the variances were unequal, the GLIMMIX procedure was used to analyze data, and the gamma distribution was chosen. The MIXED procedure was also used to conduct polynomial regression analysis in methionine requirement of mTORC1 signaling activity experiment, and residuals followed a normal distribution. It was considered significant at *P* <0.05.

## 3. Results and Discussion

### 3.1. Aggravated Oxidative Stress, Attenuated mTORC1 Activity in Developing Mammary Glands

**Figure 1:**
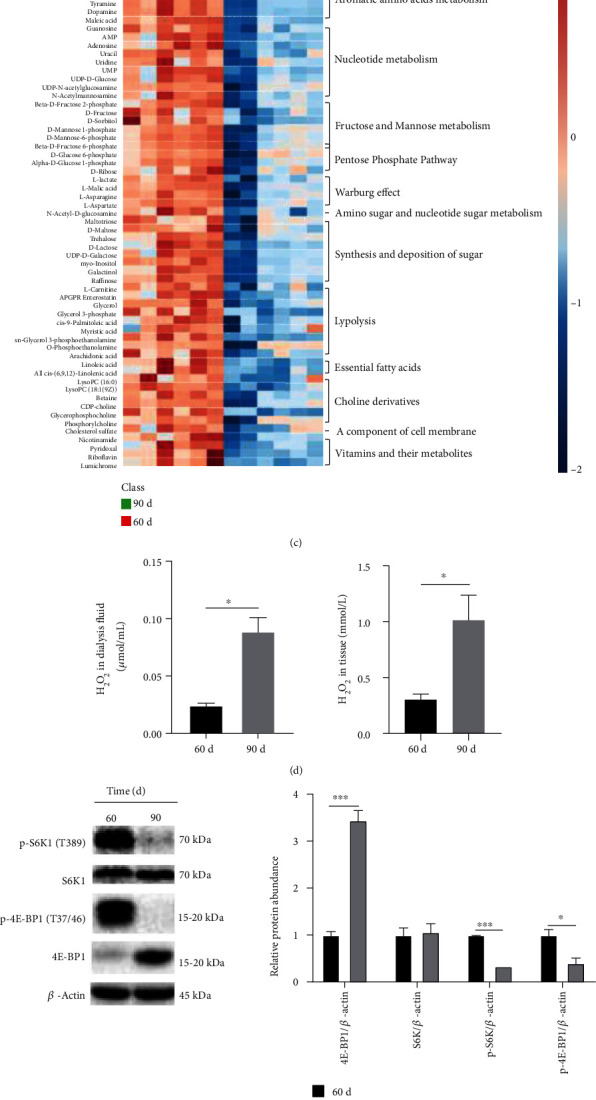
Metabolites and mTORC1 signaling in developing mammary glands at different pregnancy day. (a) The H&E stain of mammary glands at different pregnancy day. (b) Composition of mammary glands at different pregnancy day. (c) Clustering result of statistically significant metabolites shown as heat map. Distance measure using euclidean, and clustering algorithm using ward.D. (d) Hydrogen peroxide concentrations in mammary dialysis fluid and tissues. (e) The mTORC1 signaling in pregnant mammary glands. (f) Correlation heat map of statistically significant metabolites. Person's correlation was concerned in calculation. Strong correlation: 0.66-1, medium correlation: 0.33-0.66, weak correlation: 0-0.33. (g) The concentrations of S-adenosyl-L-methionine (SAM) and S-Adenosyl-L-homocysteine (SAH) in mammary glands. Values were means with SE. ∗*P* < 0.05, ∗∗*P* < 0.01, ∗∗∗*P* < 0.001.

With the advance of pregnancy, mammary glands showed extensive lobule formation (Figure 1(a)), expanded alveoli (Figure 1(a)), decreased proportion of adipocytes (Figure 1(a)) and crude fat contents (Figure 1(b)), while increased cross-sectional area, crude protein and ash contents (Figure 1(b)). Metabolomics data illustrated that mammary gland development was accompanied with promoted amino acids metabolism, thus providing major carbon and nitrogen to proliferating cells [18]. Simultaneously, there was enhanced Warburg effect required to cell proliferation [19], and increased glycogenesis, lipolysis, nucleotide metabolism, and pentose phosphate pathway (Figure 1(c)). These observations indicated mammary glands were in a rapid development stage.

Meanwhile, there was an increase in levels of 2-hydroxyadenine, formed by hydroxyl radical attack on DNA [20], in levels of glutathione disulfide (GSSG), formed by the oxidation of reduced glutathione (GSH), and in the uptake of exogenous antioxidant ergothioneine [21] (Figure 1(c)). Consistently, the relative H_2_O_2_ concentrations in mammary dialysis fluid and tissues at pregnancy day 90 was up to 3.6 and 3.3 times that at pregnancy day 60 (Figure 1(d)), respectively, suggesting disruption of redox homeostasis in developing mammary glands. Simultaneously, mammary tissues at pregnancy day 90, in comparison to that at pregnancy day 60, showed marked decrease in expression of phosphorylated S6K1 and phosphorylated 4E-BP1, the downstream of mTORC1 signaling pathway, indicating attenuated activity of mTORC1 (Figure 1(e)).

### 3.2. Increased Methionine Metabolism and SAM Requirement in Developing Mammary Glands

More methionine and its metabolites (Figure 1(c)) emerged in mammary glands at latter pregnancy time, and most metabolites had a strong correlation (correlation >0.66) with methionine (Figure 1(f), Supplementary Table 1)). The increase in *S*-methyl-5'-thioadenosine (Figure 1(c)), a vital metabolite of methionine salvage pathway, was beneficial to adenine synthesis and methionine recycle [22]. Remarkably, mammary SAM concentration at pregnancy day 90 was up to 16 times that at pregnancy day 60, whereas SAH, the initial metabolite of methionine transmethylation, remained at a relatively stable level in developing mammary glands (Figure 1(g)).

### 3.3. Hydrogen Peroxide Induced Oxidative Stress and Inhibited Cell Proliferation

**Figure 2:**
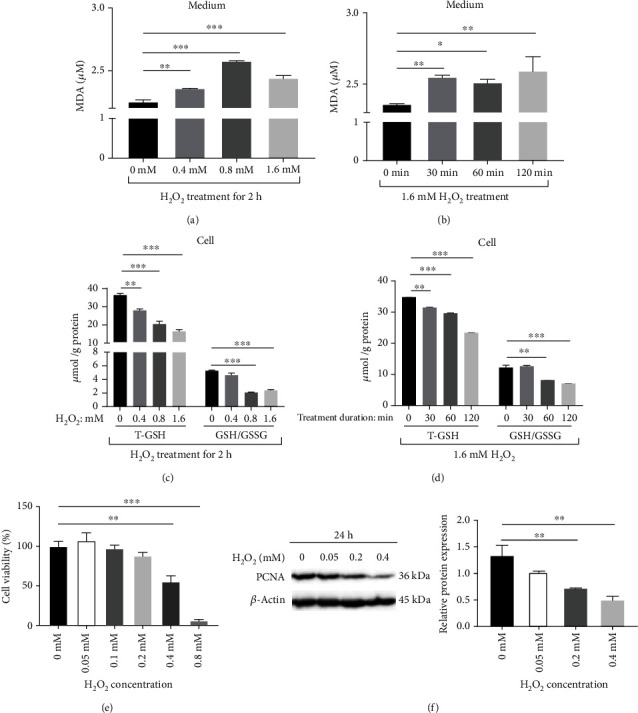
Hydrogen peroxide induced oxidative stress and inhibited cell proliferation. (a) Hydrogen peroxide increased malonaldehyde (MDA) concentrations in medium depending on the dosage of H_2_O_2_. (b) Hydrogen peroxide increased MDA concentrations in medium with time. (c) Hydrogen peroxide inhibited the balance of intracellular glutathione/glutathione disulfide (GSH/GSSG) depending on the dosage of H_2_O_2_. T-GSH: total glutathione. (d) Hydrogen peroxide inhibited the balance of intracellular GSH/GSSG with time. T-GSH: total glutathione. (e) Hydrogen peroxide dosage-dependently inhibited cell viability. (f) Hydrogen peroxide dosage-dependently inhibited cell proliferation, PCNA: proliferating cell nuclear antigen. Values were means with SE. ∗*P* < 0.05, ∗∗*P* < 0.01, ∗∗∗*P* < 0.001.

H_2_O_2_ resulted in a time- and dose-dependent increase in the production of MDA (Figure 2(a), 2(b)), a lipid peroxidation metabolite, whereas a time- and dose-dependent decrease in the total glutathione (T-GSH) and the ratio of intracellular GSH to GSSG (Figure 2(C), 2(D)), thus indicating a disruption of redox balance [23]. These results indicated that H_2_O_2_ did induce oxidative stress of mammary epithelial cells. Meanwhile, there was a decrease in cell viability (Figure 2(e)) and down-regulated protein abundance of proliferating cell nuclear antigen (PCNA) (Figure 2(f)), the marker of cell proliferation [24]. Taken together, H_2_O_2_-induced oxidative stress and depressed mammary epithelial cell survival.

### 3.4. Oxidative Stress Increased SAM Synthesis In Vitro

**Figure 3:**
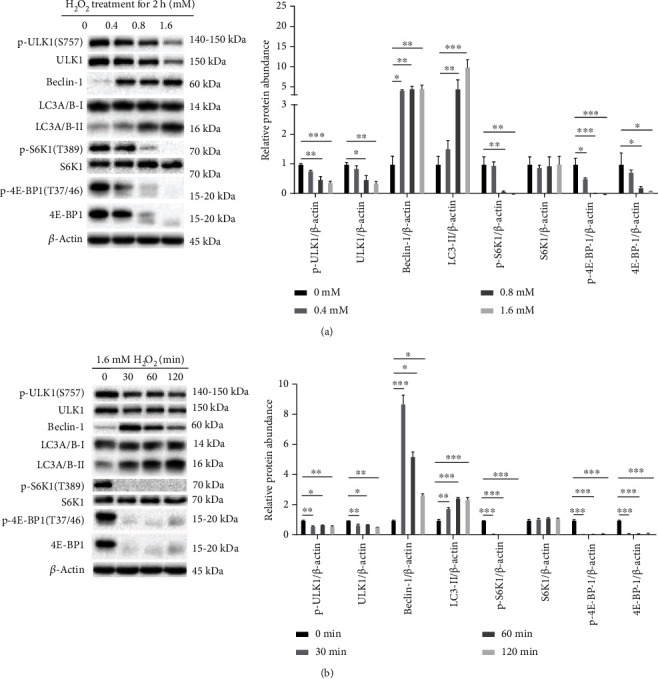
Oxidative stress inhibited mTORC1 signaling activity in mammary epithelial cells. (a) Hydrogen peroxide inhibited mTORC1 signaling pathway depending on the dosage of H_2_O_2_. (b) Hydrogen peroxide inhibited mTORC1 signaling pathway with time. Values were means with SE. ∗*P* < 0.05, ∗∗*P* < 0.01, ∗∗∗*P* < 0.001.

**Figure 4:**
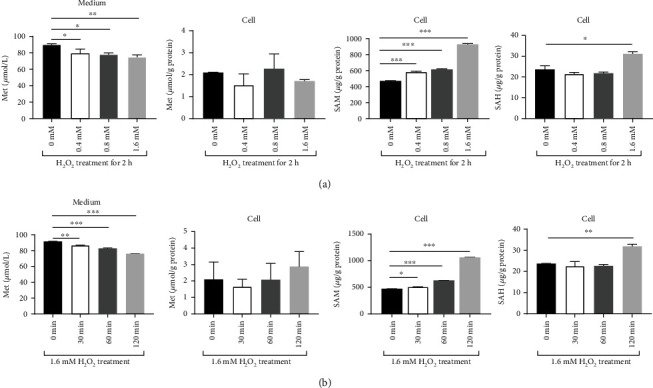
Oxidative stress increased *S*-adenosylmethionine (SAM) synthesis. (a) Hydrogen peroxide promoted SAM synthesis and reduced SAM utilization for methylation depending on the dosage of H_2_O_2_. (b) Hydrogen peroxide promoted SAM synthesis and reduced SAM utilization for methylation with time. Met: methionine. Values were means with SE. ∗*P* < 0.05, ∗∗*P* < 0.01, ∗∗∗*P* < 0.001.

H_2_O_2_ showed a dose- and time-dependence to inhibit the expression of phosphorylated ULK1 Ser 757 (p-ULK1 (S757)), p-S6K1 (T389) and phosphorylated 4E-BP1 Thr 37/46 (p-4E-BP1 (T37/46)) (Figure 3(a) and 3(b)), while promoted the expression of LC3-II and of becline-1 (Figure 3(a) and 3(b)). H_2_O_2_ promoted production of SAM, a metabolite of methionine by methionine adenosyltransferase, in a dose- and time-dependence (Figure 4(a) and 4(b)). In contrast, the increase in SAH, the metabolite of methionine by transmethylation pathway, was only observed in higher levels of H_2_O_2_ and longer time treatments (Figure 4(a) and 4(b)). These results suggested H_2_O_2_-induced oxidative stress promoted methionine utilization and reduced SAM utilization for methylation to produce SAH or T-GSH (Figures 2(c) and 2(d)).

### 3.5. Methionine Impeded H_2_O_2_-Induced Inhibition of mTORC1 Signaling Activity through SAM

**Figure 5:**
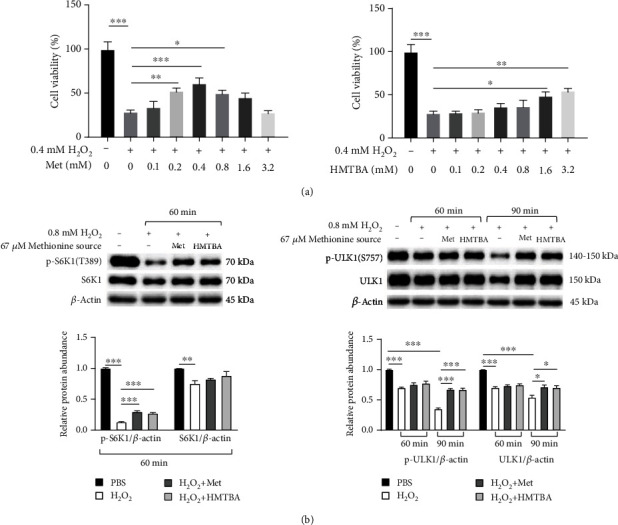
Methionine improved cell viability and reduced autophagy under oxidative stress condition. (a) L-Methionine (Met) and its hydroxyl analogue DL-2-hydroxy-4-methylthiobutanoic acid (HMTBA) relived H_2_O_2_-induced reduction of cell viability. (b) Methionine alleviated H_2_O_2_-induced inhibition of mTORC1 signaling activity. Values were means with SE. ∗*P* < 0.05, ∗∗*P* < 0.01, ∗∗∗*P* < 0.001.

**Figure 6:**
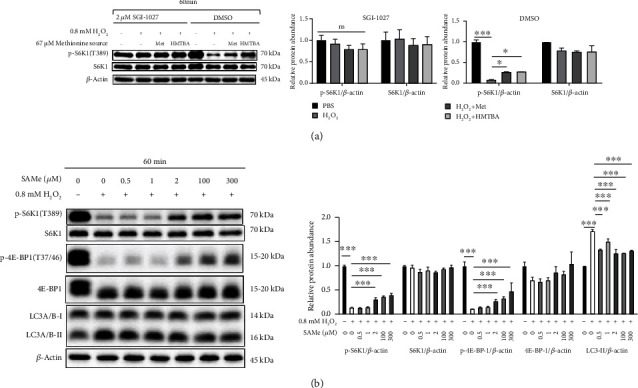
Methionine alleviated oxidative stress through SAM-activated mTORC1 independently of transmethylation. (a) Methyltransferase inhibitors mitigated H_2_O_2_-induced inhibition of p-S6K1 (T389). (b) *S*-adenosylmethionine alleviated H_2_O_2_-induced autophagy through mTORC1 signaling. SAMe: ademetionine 1,4-butanedisulfonate. Values were means with SE. ∗*P* < 0.05, ∗∗*P* < 0.01, ∗∗∗*P* < 0.001.

Methionine as well as its hydroxyl analogue, DL-2-hydroxy-4-methylthiobutanoic acid (HMTBA) that can be converted into methionine [25], increased the viability of cells exposed to H_2_O_2_ (Figure 5(a)). Furthermore, methionine showed a time dependence to promote the expression of p-S6K1 (T389) and p-ULK1 (S757) in H_2_O_2_-treated cells (Figure 5(b)). Compared with H_2_O_2_ group, cells co-cultured with H_2_O_2_, methionine or HMTBA had higher expression levels of p-S6K1 (T389), indicating that a promoted mTORC1 activity (Figure 6(a)). In the presence of SGI-1027, H_2_O_2_ would no longer change the p-S6K1 (T389) expression (Figure 6(a)). Furthermore, SAM resulted in dose-dependent activation of S6K1 and 4E-BP1, while dose-dependent downregulation of LC3-II (Figure 6(b)) in H_2_O_2_-exposed cells. Taken together, we concluded that methionine could alleviate oxidative stress-induced inhibition of mTORC1 signaling activity through SAM independent of transmethylation.

### 3.6. Discussion

It has been documented that redox imbalance, associated with oxidative stress, will rise the inaccurate mammary development [4, 5]. In this regard, it seems difficult to explain the mammary gland achieving the rapid development under the concomitant occurrence of oxidative stress, as indicated by the two critical components in redox signaling including multiplied increase in levels of H_2_O_2_, a redox signal, and markedly attenuated activity of mTORC1, a redox-signal sensor (16). Moreover, traditional wisdom mainly ascribed the antioxidant role of methionine to its transmethylation metabolites such as cysteine and GSH [26]. However, we found that oxidative stress promoted methionine utilization and reduced SAM utilization for methylation *in vivo* and *in vitro*. It implies a higher efficiency for methionine to produce SAM rather than its transmethylation products SAH or GSH to defense oxidative stress. These observations raise the possibility that methionine in physiological conditions could be independent of transmethylation to resist oxidative stress, thus helping mammary glands to achieve rapid development.

We used to recognize that the SAM activated mTORC1 by methylating PP2A [27]. Moreover, SAM participates in GSH synthesis to clear ROS, which restores oxidative stress-inhibited mTORC1 activation [26, 28]. All of the above processes require SAM to undergo transmethylation. However, under oxidative stress *in vivo* and *in vitro*, methionine utilization was increased as observed in our study, and more SAM rather than SAH and GSH was synthesized. Studies *in vitro* showed that SAM produced by methionine under oxidative stress conditions could activate the mTORC1 signaling pathway and help cells survive independent of transmethylation. Despite SAH, the initial metabolite of methionine by transmethylation pathway, was observed to increase following higher levels of H_2_O_2_ and longer time treatments *in vitro*, it showed little increase *in vivo* with the aggravation of oxidative stress as pregnancy advanced. This has an important implication in that SAM, as one of metabolites of methionine by non-transmethylation pathway, could respond earlier than methionine transmethylation pathway to oxidative signals in physiological conditions, and thus prevent the mammary gland from severe damage by more intense or chronic oxidative stress as observed *in vitro*. This might well explain the rapid development of mammary glands despite the concomitant occurrence of oxidative stress as pregnancy advanced. On the other hand, although SAH can also activate mTORC1 [10], or it takes many steps to be converted into reduced glutathione [26]. In this regard, the activation of mTORC1 by SAM represents the more direct way than by methionine transmethylation pathway. As methyltransferase inhibitor means the stronger inhibition on SAM transmethylation, the observation that methyltransferase inhibitor even impeding H_2_O_2_-inhibited p-S6K1 (T389) further indicated the significance of SAM-mediated direct activation of mTORC1 in repressing oxidative stress.

Under H_2_O_2_-induced oxidative stress, the consistent change, namely, reduced LC3-II expression while enhanced p-S6K1 (T389) expression, was observed in high concentrations of SAM treatment. It suggested that SAM might inhibit autophagy through activating mTORC1 signaling to help cell viability under oxidative stress. In contrast, little increase was observed in p-S6K1 (T389) expression though LC3-II expression was still reduced in low concentration of SAM treatment. There was evidence that mTORC1 could also inhibit autophagy by phosphorylating ULK1 on Ser 638 (p-ULK1 (S638)) [29] and phosphorylating ATG13 on Ser 258 (p-ATG13 (S258)) [30], which were not measured due to technical limitation in our study yet. Thus, a possible explanation was that p-ULK1 (S638) or p-ATG13 (S258) might be more sensitive than p-S6K1 (T389) to mTORC1 activity under oxidative stress. Nevertheless, these results could not deny that SAM activated mTORC1 signaling under oxidative stress. In support of this, cells exposed to H_2_O_2_ had enhanced p-ULK1 (S757) and p-S6K1 (T389) expression following methionine supply.

## 4. Conclusion

**Figure 7:**
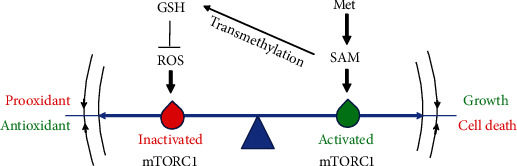
A schematic diagram of methionine protecting mammary cells against oxidative stress through producing SAM to maintain mTORC1 signaling activity.

Our study indicated that methionine protected mammary cells against oxidative stress through producing SAM to maintain mTORC1 signaling activity, thus helping cells survive and promoting mammary gland development (Figure 7). These findings have important implications for methionine nutrition in normal growth and development. On the other hand, targeting mTORC1 activity by regulation of SAM availability may be the promising interventions and preventive strategies for oxidative stress in animal and human disease.

## Data Availability

The data used to support the findings of this study are included within the article.

## Supplementary Materials

Supplementary materials Supplementary Figure 1. The isolation and purification of mammary epithelial cells. (A) Mammary epithelial cells escaped from mammary tissue block. (B) Many mammary epithelial cells escaped from mammary tissue block at 10 days after tissue block inoculation. (C) Mammary epithelial cells (left) and fibroblasts (right) grew in the medium, separately. (D) Purified mammary epithelial cells. (E) Purity identification using flow cytometry. Mammary epithelial cells accounted for more than 94 percent of the total. Supplementary Figure 2. Methionine was essential for mTORC1 signaling pathway. Mammary epithelial cells were incubated in the DMEM/F12 basic medium 2 h before the experiment. (A) Methionine starvation inhibited mTORC1 signaling but did not affect autophagy in a short time. (B) Methionine requirement of mTORC1 signaling activity. DMEM/F12 basic medium contains 115.7 *μ*M methionine. Data of methionine requirements followed linear and quadratic distribution. The optimal methionine concentration was 67 *μ*M. Values are means with SE. ∗*P* < 0.05, ∗∗*P* < 0.01, ∗∗∗*P* < 0.001. Supplementary Table 1. Pearson's correlation between methionine and other metabolites.
