# Supplementary material for: Methionine Protects Mammary Cells against Oxidative Stress through Producing S-Adenosylmethionine to Maintain mTORC1 Signaling Activity
Source: Oxid Med Cell Longev. 2021 Jul 19;2021:5550196. doi: 10.1155/2021/5550196 (PMC8315855; doi:10.1155/2021/5550196)
Supplement: Supplementary materials — Supplementary Figure 1. The isolation and purification of mammary epithelial cells. (A) Mammary epithelial cells escaped from mammary tissue block. (B) Many mammary epithelial cells escaped from mammary tissue block at 10 days after tissue block inoculation. (C) Mammary epithelial cells (left) and fibroblasts (right) grew in the medium, separately. (D) Purified mammary epithelial cells. (E) Purity identification using flow cytometry. Mammary epithelial cells accounted for more than 94 percent of the total. Supplementary Figure 2. Methionine was essential for mTORC1 signaling pathway. Mammary epithelial cells were incubated in the DMEM/F12 basic medium 2 h before the experiment. (A) Methionine starvation inhibited mTORC1 signaling but did not affect autophagy in a short time. (B) Methionine requirement of mTORC1 signaling activity. DMEM/F12 basic medium contains 115.7 μM methionine. Data of methionine requirements followed linear and quadratic distribution. The optimal methionine concentration was 67 μM. Values are means with SE. ∗P < 0.05, ∗∗P < 0.01, ∗∗∗P < 0.001. Supplementary Table 1. Pearson's correlation between methionine and other metabolites. [file 5550196.f1.docx]

Supplementary Materials


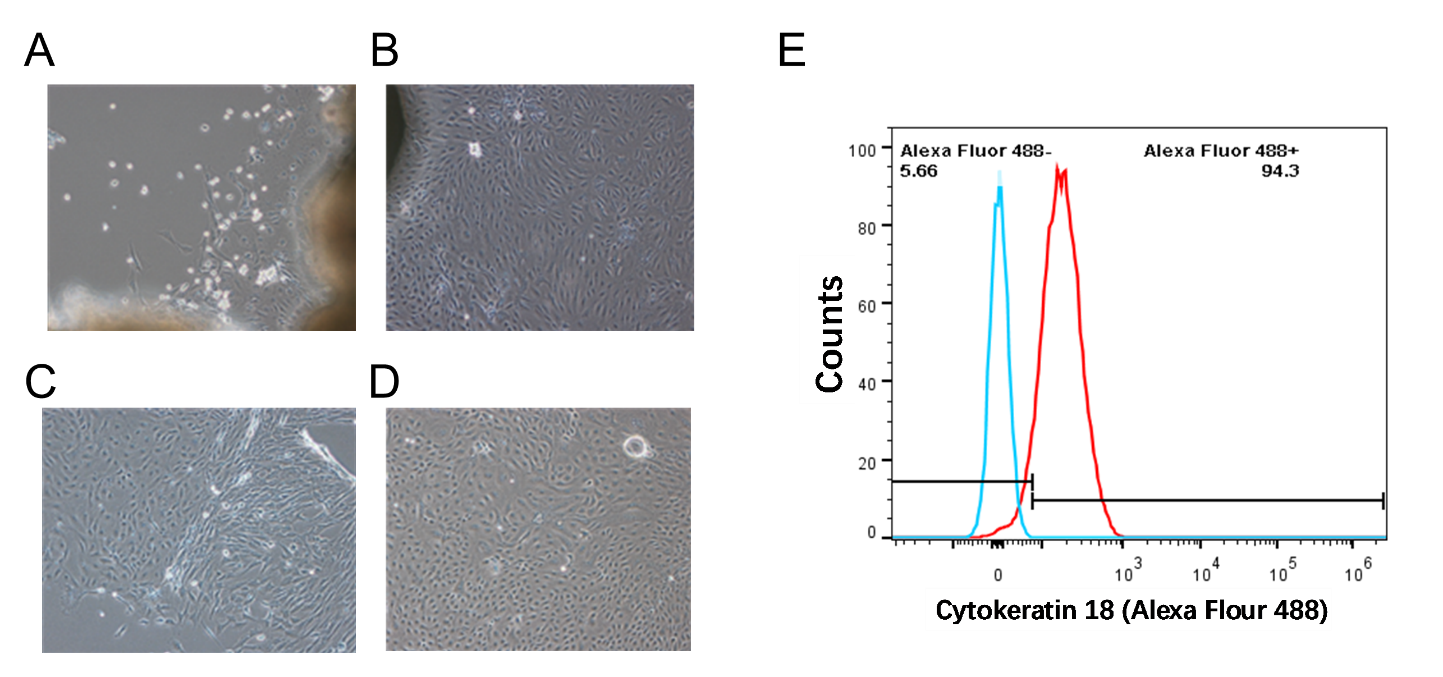


Supplementary Figure 1. The isolation and purification of mammary epithelial cells. (A) Mammary epithelial cells escaped from mammary tissue block. (B) Many mammary epithelial cells escaped from mammary tissue block at 10 days after tissue block inoculation. (C) Mammary epithelial cells (left) and fibroblasts(right) grew in the medium, separately. (D) Purified mammary epithelial cells. (E) Purity identification using flow cytometry. Mammary epithelial cells accounted for more than 94 percent of the total.


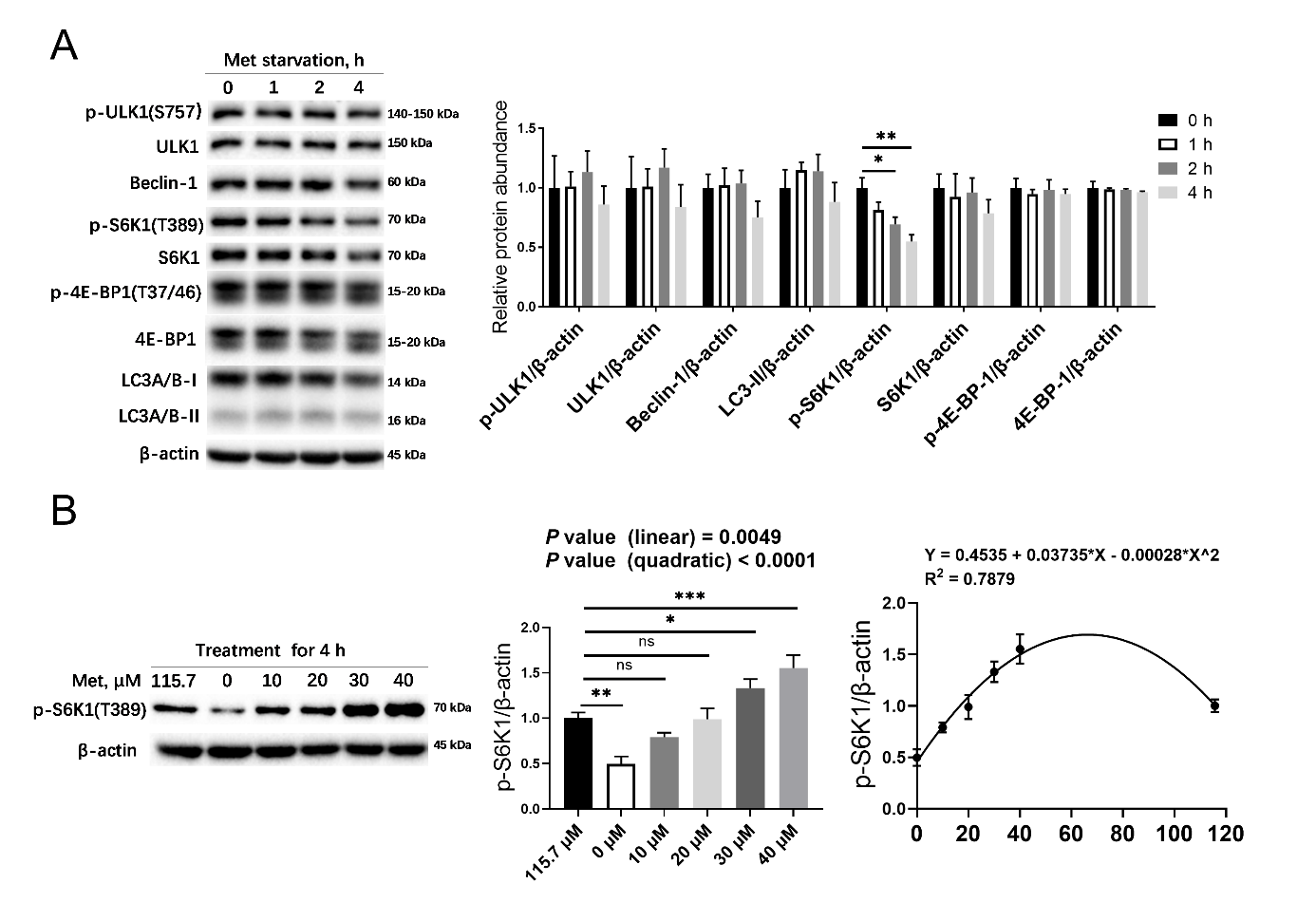


Supplementary Figure 2. Methionine was essential for mTORC1 signaling pathway. Mammary epithelial cells were incubated in the DMEM/F12 basic medium 2 h before the experiment. (A) Methionine starvation inhibited mTORC1 signaling but did not affect autophagy in a short time. (B) Methionine requirement of mTORC1 signaling activity. DMEM/F12 basic medium contains 115.7 μM methionine. Data of methionine requirements followed linear and quadratic distribution. The optimal methionine concentration was 67 μM. Values were means with SE. * *P* < 0.05, ** *P* < 0.01, *** *P* < 0.001.

Supplementary Table 1. Pearson’s correlation between methionine and other metabolites.

| Metabolites | Correlation | *P* |
| --- | --- | --- |
| L-Methionine | 1 | 0 |
| L-Leucine | 0.98589 | 4.30E-09 |
| Betaine | 0.98579 | 4.45E-09 |
| L-Phenylalanine | 0.98525 | 5.37E-09 |
| myo-Inositol | 0.98195 | 1.46E-08 |
| Tyramine | 0.97856 | 3.44E-08 |
| Ergothioneine | 0.97777 | 4.12E-08 |
| L-Isoleucine | 0.97735 | 4.52E-08 |
| L-Tyrosine | 0.97718 | 4.69E-08 |
| Dopamine | 0.97651 | 5.41E-08 |
| L-Valine | 0.9746 | 7.98E-08 |
| Guanosine | 0.9724 | 1.20E-07 |
| Raffinose | 0.96782 | 2.57E-07 |
| L-Pyroglutamic acid | 0.96594 | 3.41E-07 |
| L-Glutamine | 0.96246 | 5.51E-07 |
| L-Tryptophan | 0.95813 | 9.45E-07 |
| 5-L-Glutamyl-L-alanine | 0.95724 | 1.05E-06 |
| Maleic acid | 0.95594 | 1.21E-06 |
| L-Malic acid | 0.95547 | 1.28E-06 |
| Galactinol | 0.95114 | 2.02E-06 |
| Beta-D-Fructose 2-phosphate | 0.95072 | 2.11E-06 |
| UDP-D-Galactose | 0.95032 | 2.19E-06 |
| Hypoxanthine | 0.94925 | 2.43E-06 |
| alpha-D-Glucose 1-phosphate | 0.94898 | 2.50E-06 |
| D-Mannose-6-phosphate | 0.94758 | 2.85E-06 |
| L-Aspartate | 0.94729 | 2.93E-06 |
| Cytidine 5'-diphosphocholine | 0.94579 | 3.37E-06 |
| Trehalose | 0.9446 | 3.74E-06 |
| L-Alanine | 0.94167 | 4.82E-06 |
| Argininosuccinic acid | 0.94059 | 5.27E-06 |
| L-Asparagine | 0.93991 | 5.57E-06 |
| Serine | 0.93779 | 6.61E-06 |
| Allopurinol riboside | 0.93477 | 8.33E-06 |
| Uridine | 0.93415 | 8.72E-06 |
| Beta-D-Fructose 6-phosphate | 0.93365 | 9.05E-06 |
| N-Acetylmannosamine | 0.93335 | 9.25E-06 |
| Uracil | 0.93233 | 9.97E-06 |
| D-Mannose 1-phosphate | 0.93153 | 1.06E-05 |
| UDP-N-acetylglucosamine | 0.92978 | 1.19E-05 |
| Inosine | 0.92721 | 1.42E-05 |

Strong correlation: 0.66-1, medium correlation: 0.33-0.66, weak correlation: 0-0.33. It was considered significant at *P* < 0.05.

Supplementary Table 1. Pearson’s correlation between methionine and other metabolites. (continued)

| Metabolites | Correlation | *P* |
| --- | --- | --- |
| Uridine diphosphate glucose | 0.92663 | 1.48E-05 |
| Lactate | 0.92213 | 1.98E-05 |
| Uridine 5'-monophosphate | 0.91728 | 2.65E-05 |
| 2-Hydroxyadenine | 0.91663 | 2.75E-05 |
| APGPR Enterostatin | 0.90614 | 4.89E-05 |
| D-Lactose | 0.90469 | 5.27E-05 |
| D-Ribose | 0.90263 | 5.84E-05 |
| D-Proline | 0.9019 | 6.06E-05 |
| Adenosine monophosphate | 0.90111 | 6.30E-05 |
| Phosphorylcholine | 0.89415 | 8.74E-05 |
| D-Glucose 6-phosphate | 0.88697 | 0.0001199 |
| Nicotinamide | 0.88558 | 0.0001271 |
| D-Maltose | 0.88419 | 0.0001346 |
| Glycerol | 0.88277 | 0.0001428 |
| Glutathione disulfide | 0.88122 | 0.000152 |
| Vitamin B_6_ | 0.8811 | 0.0001528 |
| N-Acetyl-D-glucosamine | 0.87873 | 0.0001679 |
| L-Carnitine | 0.87634 | 0.0001844 |
| S-Methyl-5'-thioadenosine | 0.8747 | 0.0001964 |
| O-Phosphoethanolamine | 0.87108 | 0.000225 |
| Adenosine | 0.86525 | 0.0002778 |
| sn-Glycerol 3-phosphoethanolamine | 0.86157 | 0.0003158 |
| Arachidonic Acid | 0.85514 | 0.0003918 |
| Cholesterol sulfate | 0.84735 | 0.0005022 |
| Riboflavin | 0.84698 | 0.000508 |
| Taurine | 0.83781 | 0.0006686 |
| D-Fructose | 0.83666 | 0.0006912 |
| Linoleic acid | 0.82235 | 0.0010256 |
| Maltotriose | 0.82 | 0.0010905 |
| Lumichrome | 0.80861 | 0.0014522 |
| 2-Pyrrolidineacetic acid | 0.801 | 0.001741 |
| LysoPC1819Z | 0.78527 | 0.002475 |
| all cis-6,9,12-Linolenic acid | 0.78168 | 0.0026718 |
| D-Sorbitol | 0.76908 | 0.0034569 |
| cis-9-Palmitoleic acid | 0.76521 | 0.0037296 |
| Glycerophosphocholine | 0.722 | 0.0080154 |
| Myristic acid | 0.71987 | 0.0082937 |
| Glycerol 3-phosphate | 0.70852 | 0.0099035 |
| LysoPC160 | 0.64154 | 0.024535 |
| L-Arginine | 0.62096 | 0.031169 |

Strong correlation: 0.66-1, medium correlation: 0.33-0.66, weak correlation: 0-0.33. It was considered significant at *P* < 0.05.
